# ATF4: Orchestrating Cellular Stress Adaptation, Metabolism, and Immune Regulation in Health and Disease

**DOI:** 10.3390/ijms27093784

**Published:** 2026-04-24

**Authors:** Chunyan Wang, Fengjing Jia, Feng He

**Affiliations:** The Center for Cancer Research, School of Integrative Medicine, Shanghai University of Traditional Chinese Medicine, Shanghai 201203, China; wangcyxb@163.com

**Keywords:** ATF4, integrative stress response, endoplasmic reticulum, unfolded protein response

## Abstract

Activating transcription factor 4 (ATF4) is a master transcription factor of integrated stress response (ISR), an evolutionarily conserved intracellular signaling network that helps the cell, tissue, and organism to adapt to various unpredictable environmental fluctuations, mitigate the challenges, and maintain health. Stress-induced ATF4 expression regulates a wild variety of gene expression programs to enable stress management and repair for cell homeostasis and integrity. However, chronic ATF4 activation contributes to pathologies including cancer, inflammation, and neurodegeneration. Extensive studies have revealed that ATF4 regulates many cellular processes including autophagy, apoptosis, metabolism, and inflammation. Emerging evidence has uncovered new signaling pathways in regulation of ATF4 expression and activation, including at transcriptional, translational, and post-translational levels, and new functions of ATF4 in the progression of various metabolic and stress-related diseases, including inflammation, cancer, and cardiovascular disease. The diversity of ATF4 functions is increasingly appreciated. This review summarizes the recent findings of the complex regulatory network of ATF4 activity and its roles in integrating stress responses, metabolic reprogramming, unfolded protein responses, autophagy, inflammation, and immunity.

## 1. Introduction

Mammalian cells are constantly exposed to a plethora of exogenous and endogenous stressors that can disrupt organelle and cell functions, triggering different stress response mechanisms to determine cell adaptation, cell fate, and their functional identities [1,2,3,4,5]. These stress response pathways, involving a series of signaling transduction and gene expression programs, often temporarily shift energy and resources from normal cellular processes toward stress management for survival and homeostasis. Activating transcription factor 4 (ATF4), a master regulator of the endoplasmic reticulum (ER) stress and integrated stress response (ISR), plays a pivotal role in balancing survival and stress adaptation [6,7,8]. ATF4 belongs to the ATF/cAMP-response element binding protein (CREB) family (named after the ATF site and CRE regulatory sequences in gene promoters to which these proteins bind) of basic leucine zipper (bZip) transcription factors, which comprises ATF1-7, cAMP-responsive element modulator (CREM), CREB3L4, and CREB-H [9,10]. These members form homo- or heterodimers to recognize and bind to CRE and other specific response elements, thereby inducing transcription of various target genes and contributing to intracellular homeostasis. Under normal physiological conditions, ATF4 expression is very low with a basal half-life of <1 h, controlled by the rates of its transcription, translation, and proteosome-mediated degradation [11]. The basal transcript levels of ATF4 display tissue-specific patterns, with elevated expression in metabolically active tissues such as skeletal muscle, hypothalamus, and kidney [12,13,14]. However, its half-life is often altered under various stress conditions to mediate cell adaptations. For instance, ATF4’s half-life is shortened in chemoresistant gastric cancer cells compared to sensitive cells [15]. In the reoxygenated anoxic HeLa cells, ATF4 half-life is shorter than 5 min [16]. Proteasome inhibitors like bortezomib (BTZ) or MG132 stabilize ATF4, extending its half-life [17,18]. Glucose or glutamine deprivation significantly prolongs the half-life of *ATF4* mRNA by inhibiting m^6^A modification and YTHDF2-mediated RNA decay to induce autophagy for cell survival under glutaminolysis inhibition [19]. However, the upregulation of ATF4 proteins levels is mainly regulated by stress-induced transcription and selective translation mediated by phosphorylation of the eukaryotic translation initiation factor 2 subunit alpha (eIF2α-p)-canonical ISR pathway through four stress-sensing kinases: general control non-derepressible 2 (GCN2), PKR-like ER kinase (PERK), heme-regulated inhibitor (HRI), and protein kinase R (PKR) [4]. A recent study revealed that split ISR, when eIF2B activity is decreased in absence of eIF2α-p induction, can also upregulate ATF4 protein levels by eIF4E-dependent translation of the upstream open reading frame 1 (uORF1) and subsequent stabilization of *ATF4* mRNA [20]. Upregulated ATF4 subsequently translocates to the nucleus and forms homodimers and heterodimers with other bZIP proteins that trigger widespread transcriptional changes to restore cellular homeostasis [21]. ATF4-induced target genes participate in the regulation of apoptosis, ferroptosis, autophagy, metabolism, mitochondrial biogenesis, inflammation, and immunity, that collectively mediate unfolded protein responses and integrated stress response. Once the stressor is mitigated, ATF4 protein is rapidly degraded and these signaling pathways are deactivated, enabling cells to resume their identities and functional integrity for normal biological processes. If the stress is prolonged or too severe, ATF4 mediates cell death to remove the diseased/stressed cells to limit the spread of injury and maintain organism homeostasis. The temporal regulation of ATF4 transcription, translation, and degradation allows the cells to fine-tune ATF4 activity that efficiently initiate and terminate the sophisticated cell response mechanisms to resolve the stress, thereby ensuring the survival and adaptation of organisms.

However, ATF4 displays the “Dr. Jekyll and Mr. Hyde” effect on health and disease. Transient ATF4 expression and activation mediate a protective mechanism in normal cells encountering the acute stress [6]. Prolonged ATF4 expression and activation under chronic stress promote tumor transformation [6,22]. Cancer cells can hijack the ATF4-mediated stress response programs to enable cancer cell proliferation, metastasis, and chemoresistance [22,23,24]. Emerging studies expanded our understandings from transient and selective activation of ATF4-mediated cellular stress response programs to new roles in the regulation of biological processes and implications in disease development. In this review, we provide an overview of ATF4, including its structure, expression regulation, and functions, with a focus on the recent findings on the complex regulatory network of ATF4 activity and its roles in integrating stress responses, metabolic reprogramming, unfolded protein responses, autophagy, mitochondrial biogenesis, inflammation, and immunity.

## 2. Protein Structure of ATF4

**Figure 1:**
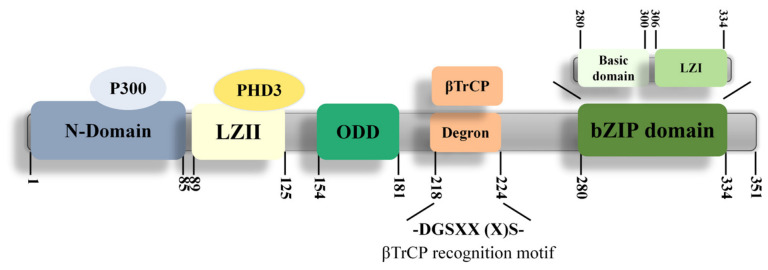
Schematic representation of the domain organization of ATF4 protein. Structure of ATF4 with N-terminal domain (interaction with P300), LZII motif (interaction with PHD3), ODD domain, βTrCP recognition motif, and bZIP domain.

Human ATF4 protein has 351 amino acids with multiple conserved domains/motifs that collectively affect ATF4 activity. The bZIP domain at the C-terminus is responsible for DNA binding and protein dimerization. It contains basic domain (BD) that binds to specific DNA sequences such as CREs and amino acid response elements (AAREs), and leucine zipper I (LZI) motif that forms homo- or hetero-dimers with other bZIP domain-containing proteins for transcription regulation [21,25,26]. Meanwhile, the leucine zipper II (LZII) motif interacts with prolyl-4-hydroxylase domain 3 (PHD3) to regulate ATF4 stability [27]. Oxygen-dependent degradation (ODD) domain mediates part of hypoxia-induced ATF4 activation, exerting specific gene therapeutic effects by post-translational regulation of ATF4 [27,28]. The DSGXX(X)S motif, when phosphorylated, is recognized and bound by the E3 ligase β-transducin repeat-containing protein (βTrCP), hence termed the phosphodegron DpSGXX(X)pS, leading to polyubiquitination and proteasome-mediated degradation of ATF4. The N-terminal domain, known as the transcriptional activation site, binds directly to histone acetyltransferase p300, which then recruits ATF4 into the nuclear speck to sequester ATF4 from binding to βTrCP and increases ATF4 stability. p300/CBP-associated factor (PCAF) induces bZIP acetylation, and inhibits ATF4 ubiquitination and degradation, increasing ATF4 transcriptional activity [29,30,31]. These domains/motifs regulate ATF4 stability, DNA binding capacity, and dimerization levels in various ways to affect ATF4-mediated transcriptional programs in response to various pathological injuries (Figure 1).

## 3. Post-Transcriptional Regulation of ATF4

**Figure 2:**
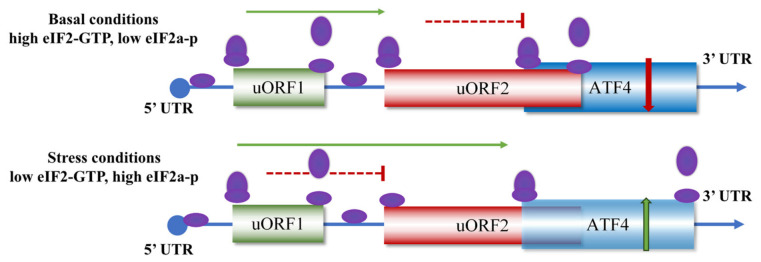
Translational regulation of *ATF4* mRNA by uORFs. Basal conditions (**upper panel**): when cellular levels of the translation initiation factor eIF2-GTP are abundant, a scanning ribosome (purple) efficiently translates uORF1 and remains competent for re-initiation. It subsequently re-acquires a ternary complex and re-initiates translation at uORF2. Since uORF2 overlaps ATF4, its translation leads to ribosome dissociation at the uORF2 stop codon, thereby repressing ATF4 protein synthesis. Stress conditions (**lower panel**): upon cellular stresses (ER stress, amino acid deprivation), phosphorylation of eIF2α reduces global eIF2-GTP availability. While the ribosome still translates uORF1, the delayed re-acquisition of a new ternary complex prevents efficient re-initiation at uORF2. This allows a fraction of ribosomes to bypass the uORF2 start codon, continue scanning, and instead initiate translation at the authentic ATF4 AUG start site, leading to a marked upregulation of ATF4 protein expression.

The mammalian *ATF4* gene generates two distinct mRNA transcript variants, referred to as the long and short isoforms, which differ primarily in their 5′untranslated region (5′UTR) [32]. The long transcript variant contains a complex 5′UTR with multiple uORFs, including uORF1 and uORF2, which overlaps with the ATF4 ORF. These uORFs are crucial for ATF4’s stress response. In the traditional delayed translation reinitiation model, ORF1 is a positive-acting element that facilitates ribosome scanning and downstream translation reinitiation. uORF2 acts as a potent inhibitory element that suppresses ATF4 translation. Under basal conditions with sufficient eIF2·GTP and a low level of eIF2α-p, the assembled 40S ribosome resumes scanning after translation of uORF1 and acquires a new eIF2·GTP·Met-tRNAiMet pre-initiation complex to reinitiate translation at the uORF2 initiation codon, leading to translation termination and ribosome dissociation at the stop codon of uORF2, out of frame of the ATF4 coding sequence [28]. Under cellular conditions when eIF2·GTP is insufficient and eIF2α-p level is upregulated, the insufficient delivery of eIF2·GTP·Met-tRNAi to 40S ribosome delays reinitiating complex formation. Consequently, after completion of uORF1 translation, the scanning 40S ribosomal subunit passes through uORF2 and reinitiates at the start codon of ATF4, resulting in elevated ATF4 translation [33]. In contrast, the short transcript variant arises from alternative promoter usage or splicing and lacks uORF2. This structural simplification enables ribosomes to bypass inhibitory elements more efficiently, resulting in significantly higher translational efficiency than the long variant. Notably, expression levels of the two variants are not static. Under normal physiological conditions, the long variant may constitute the predominant form. However, upon exposure to various cellular stresses, notably amino acid deprivation, ER stress, and oxidative stress, the short transcript variant is frequently upregulated and selectively utilized. This stress-induced shift ensures rapid ATF4 protein production, critical for initiating ISR [34,35] (Figure 2).

Recent studies revealed that a highly conserved stem-loop in the uORF2/ATF4 overlap enables ribosome queuing to provide another layer of ATF4 translational control, which explains how the inhibitory uORF2 can be translated under stress, contradicting the original delayed translation reinitiation model [36]. Interestingly, Maria Hatzoglou et al. reported a new regulatory mechanism of ATF4 translation triggered by decreased eIF2B activity in the absence of eIF2α-p induction, termed split-ISR, in which eIF4E mediates the translation of uORF1 and subsequent stabilization of *ATF4* mRNA [20]. Split-ISR plays an important role in the in human diseases, including leukodystrophies, and it is highly dependent on uORF1 but eIF2α-p independent. ATF4 translational control is more complex than originally describedp; its key role in diverse biological processes and its regulatory mechanism needs further investigation.

Beyond transcriptional and uORF-mediated translational control, ATF4 expression is further modulated post-transcriptionally by non-coding RNAs. Specific microRNAs (miRNAs), including *miR-214*, *miR-1283*, *miR-383-3p*, *miR-193a-3p*, *miR-93-3p*, *miR-129/342*, *miR-15b-5p*, *miR-3200-5p*, *miR-663*, *miR-320a*, and *miR-4734*, directly/indirectly target ATF4 to induce mRNA degradation or repress translation in certain contexts (Table 1). Long non-coding RNAs (lncRNAs) such as *MALAT1* and *HOTAIR* have been reported to modulate ATF4 expression through post-transcriptional mechanisms [37]. Additionally, DAMER positively regulates ATF4. Upon glucose or glutamine deprivation, DAMER is induced and undergoes m^6^A demethylation, retaining in the nucleus. It then directs the m^6^A demethylase ALKBH5 to bind to *ATF4* mRNA, removes its m^6^A modification, and enhances both the stability and translational efficiency of *ATF4* mRNA [38]. These RNA-based layers of regulation collectively ensure tight spatial and temporal control of ATF4 activity, complementing the isoform-specific dynamics described above.

ATF4 stability is tightly dependent on SCFβTrCP E3 ubiquitin ligase, which recognizes ATF4 phosphorylated at serine 219 (S219) within DSGXX(X)S degradation motif [11,52,53]. βTrCP recognizes the accumulation of additional negative charges in the region around the motif and further promotes the interaction with ATF4 by phosphorylating threonine 213 (T213), S224, S231, S235 and S248, resulting in ATF4 destabilization [54]. Casein kinase 1δ (CK1δ) is a potential kinase controlling the phosphorylation of ATF4-S219, which promotes βTrCP-mediated polyubiquitination and proteasomal degradation [15]. CK1δ activity is mainly mediated by autophosphorylation within the C-terminal structural domain. Protein kinase A (PKA), protein kinase B (PKB/Akt), protein kinase Cα (PKCα), checkpoint kinase 1 (Chk1), cdc-like kinase 2 (CLK2), cytokinin-dependent kinase 2/cytokinin E (CDK2/E), and cytokinin-dependent kinase 5/p35 (CDK5/p35) phosphorylate specific residues at the relevant sites of CK1δ, inhibit CK1δ activity, and improve ATF4 stability by reducing βTrCP-mediated ubiquitination and degradation [55,56,57]. Additionally, p300 stabilizes ATF4 by interfering with βTrCP interactions, while hypoxia-inducible PHD3 enhances stability via proline hydroxylation in ODD domain [27]. ATF4 transcription is mediated by different transcription factors: protein arginine methyltransferase 5 (PRMT5) inhibition results in ATF4 with unstable, intron-retaining nuclear isoform, downregulating ATF4 transcription [58]; NRF2 activates ATF4 transcription by directly binding to the antioxidant response element (ARE) sequence in ATF4 promoter region under oxidative stress conditions [6,59]; CLOCK regulates ATF4 expression under cisplatin and etoposide [60,61]; pancreatic and duodenal homeobox 1 (PDX1) and ATF4 form stress responsive complexes and co-occupy C/EBP-ATF response elements (CARE) sites [62]. The intricate interplay between ATF4’s stability and transcriptional regulation positions it as a central coordinator of cellular stress responses. Thus, depth-understanding of ATF4 provides insights into both physiological adaptation and pathological states.

## 4. Regulation of Transcriptional Activation of ATF4 Target Genes

**Figure 3:**
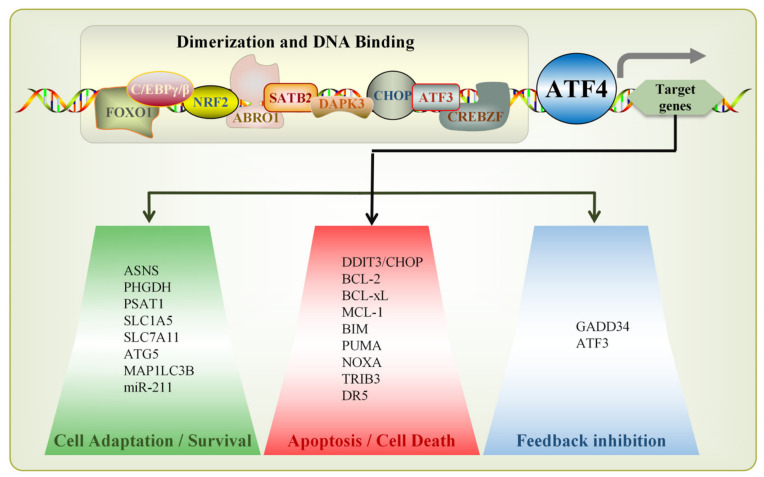
Transcriptional regulatory network orchestrated by ATF4. At the top, the “Dimerization and DNA Binding” segment illustrates diverse transcription factors involved in homodimerization, heterodimerization, or DNA-binding processes. ATF4 acts as a central transcriptional regulator, driving the expression of distinct subsets of target genes, which are functionally partitioned into three categories: cell adaptation/survival, apoptosis/cell death, and feedback inhibition.

ATF4 mainly regulates target genes through dynamic dimerization mediated by its leucine zippers (LZs). LZs are amphiphilic structural motifs that form helical dimers with LZs of the same or different bZIP proteins, such as CHOP/abraxas brother 1 (ABRO1)/special AT-rich sequence-binding protein 2 (SATB2)/death-associated protein kinase 3 (DAPK3)/, enabling both homo- and hetero-dimerization [63,64,65]. This dimerization preference underpins ATF4’s transcriptional regulatory versatility (Figure 3). During ER stress, ATF4 promotes the transcription of UPR target gene DNA damage-inducible transcript 3 (*DDIT3*, also known as *CHOP*), which further downregulates the transcription of genes encoding the mitochondrial anti-apoptotic proteins, such as B-cell lymphoma-2 (*BCL-2*)/*BCL-xL*/myeloid cell leukemia-1 (*MCL-1*) and upregulates BCL-like 11 (*BIM*) expression, thereby mediating apoptosis [66,67,68]. ATF4 also forms heterodimers with other bZIP factors: it partners with CCAAT/enhancer-binding proteinγ (*C/EBPγ*, a key stress-responsive C/EBP family member) to co-bind CARE and co-regulate ISR-related genes [69,70]; collaborates with *ATF3* (a rapid stress/hypoxia-induced ISR component) to modulate apoptosis and biosynthesis pathways via the ATF3-ATF4 stress axis [71]; and dimerizes with *NRF2* at ARE to induce heme oxygenase-1 (HO-1) expression [72]. Beyond dimerization, ATF4 responds to ER stress by activating enzymes for antioxidant defense and amino acid metabolism, as well as inducing growth factors and molecular chaperones to regulate target genes, such as *DDIT3/CHOP*, *GADD34*, asparagine synthetase (*ASNS*) and solute carrier family 7 member 11 (*SLC7A11*). The tribbles pseudokinase 3 (TRIB3), which normally regulates NF-κB-induced gene expression under non-stress conditions, shifts to suppress ATF4 after ER stress. It binds the C/EBP-ATF complex, co-localizes with ATF4, and promotes ATF4 degradation while repressing the transcriptional activities of ATF4, DDIT3, and C/EBPβ. TRIB3 overexpression suppresses ATF4 activity, resulting in increased HepG2 cell viability and delayed cell death, indicating increased cellular resistance to BTZ [67]. BTZ, the first approved proteasome inhibitor, is used for multiple myeloma and mantle cell lymphoma [73]. Context-dependent roles further highlight complexity: low ATF4 expression was detected in both clinical samples and resistant sublines, while high expression of ATF4 was found to significantly reverse BTZ resistance in osteosarcoma (OS)/BTZ cells by activating CBL proto-oncogene C (CBL-c) transcription and accelerating RET degradation [74]. In addition, dihydroartemisinin (DHA)-induced ER stress upregulates ATF4, which increases heat shock protein family A member 5 (HSPA5) expression. HSPA5 then enhances glutathione peroxidase 4 (GPX4) activity, triggering an anti-peroxidative response that protects glioma cells from ferroptosis [75].

The question that remains to be further investigated is whether the diverse dimerization of ATF4, which possesses a wide variety of regulatory mechanisms, influences specific subgroups of ATF4 target genes with high probability. For example, C/EBPβ, an ATF4 heterodimeric transcription factor induced by a wide range of extracellular stimuli, is involved in numerous biological processes, including gluconeogenesis, adipogenesis, hematopoiesis, hepatic regeneration, and apoptosis by regulating the expression of the corresponding target genes. However, higher amounts of ATF4 may saturate TFs, which may alter or inhibit the actions of these transcription factors [76,77,78]. Therefore, the role of the relative amount of homo- and hetero-dimerizations on the regulation of target genes remains to be further explored.

## 5. ATF4 Regulation of Integrated Stress Response

**Figure 4:**
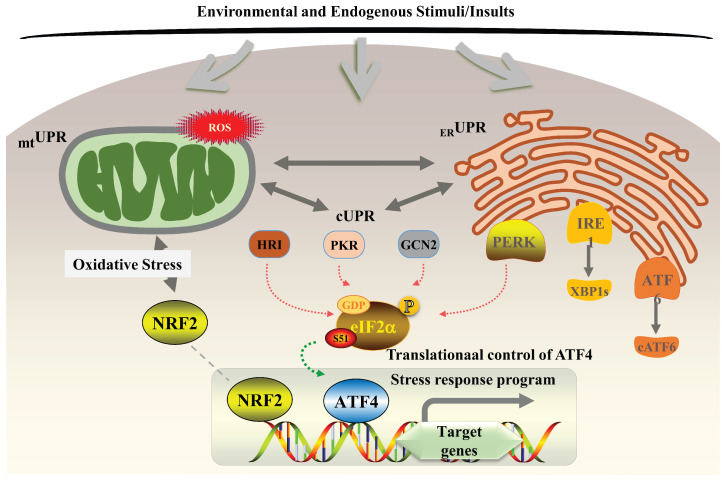
The ISR and UPR networks. Various stressors, including toxins, metabolic/nutrient imbalances, oxidative stress (ROS/RNS), and organelle (mitochondrial/ER) stress, activate specific upstream kinases (HRI, PKR, GCN2, PERK). These kinases converge on a central regulatory node by phosphorylating the alpha subunit of eIF2α at S51. This phosphorylation event attenuates global protein synthesis while selectively promoting the translation of key transcription factors like ATF4. Simultaneously, distinct stress sensors are activated, leading to the production of transcription factors such as XBP1s and cATF6. These transcription factors, along with NRF2 and ATF4, coordinately induce a stress-adaptive transcriptional program. This program upregulates genes (*CHOP*, *C/EBPs*, *ATF3*, *TRIB3*) involved in metabolism, autophagy, and protein folding, which collectively determine cell fate. The output of this integrated network (_mt_UPR, _ER_UPR, cUPR) influences critical cancer hallmarks, including cell survival, proliferation, metabolism, and metastasis, highlighting its role in pathophysiology.

ISR is a cellular adaptive reaction triggered by various stressors, centered on the phosphorylation of eIF2α. Its key mechanism involves four upstream kinases, including PERK/GCN2/PKR/HRI, that sense distinct stress signals and converge on the phosphorylation of the α subunit of eIF2α at S51, thereby inhibiting global protein synthesis and selectively inducing the expression of specific genes (such as ATF4) to promote cellular recovery [79,80,81,82] (Figure 4). PERK is activated by the accumulation of misfolded proteins in ER, and its phosphorylation of eIF2α indirectly inhibits the synthesis of nascent polypeptides and reduces their entry into the ER lumen [83,84]; GCN2 is primarily activated in response to amino acid deprivation (via uncharged tRNA accumulation) [85]; PKR is mainly activated by viral infection (via double-stranded RNA), blocking viral mRNA translation and promoting apoptosis, and can also be activated by oxidative stress, ER stress, cytokine signaling, and growth factors [86,87,88]; HRI is activated by low heme levels (such as hemolytic anemia, heme synthesis disorders) and directly phosphorylates eIF2α [89]. Phosphorylation of eIF2α reduces the concentration of the eIF2 ternary complex, thereby inhibiting global protein synthesis and upregulating ATF4 expression to restore cellular viability. Currently, several eIF2α kinase activators can enhance ISR signaling: BTZ, gemcitabine, and CCT020312 for the PERK pathway [90,91,92]; halofuginone and arginine deiminase (mimicking amino acid deprivation) for the GCN2 pathway [93,94,95]; BEPP for the PKR pathway [96]; and BTdCPU for the HRI pathway [97]. These compounds exert anticancer effects by promoting eIF2α phosphorylation to stably upregulate ATF4 expression.

ATF4 plays a dual role in ISR. On the one hand, for pro-survival effects, ATF4 binds to anti-apoptotic protein *MCL-1* at position −332 to −324 [98]; it also upregulates the transcription factor nuclear protein 1 (NUPR1), which regulates DNA repair-related genes and exerts an anti-apoptotic effect [99]. In addition, ATF4’s target gene *miR-211* has a pro-survival effect. The PERK-eIF2α-ATF4 axis rebuilds cellular homeostasis before apoptosis by inhibiting the expression of the pro-apoptotic factor CHOP [100]. Conversely, for pro-apoptotic effects, when stress persists and the cell fails to restore homeostasis, ATF4 transcriptionally activates pro-apoptotic genes encoding *CHOP*, *TRIB3*, etc., leading to cell death [101]. ATF4 also regulates the pro-apoptotic protein NOXA at the transcriptional level and triggers the intrinsic apoptosis pathway by adjusting the expression of pro- and anti-apoptotic BCL-2 family members [102]. Notably, in the context of CHOP-dependent stress, the death receptor 5 (DR5) mediates apoptosis independently of ATF4 via FADD and caspase-8 [103]. Therefore, ISR regulates key signaling pathways through ATF4, playing a dual role in cancer: it can both promote tumor cell adaptation and survival and direct cell death.

## 6. ATF4 Regulation of Unfolded Protein Response

The UPR is coordinated by three major sensors residing in the ER membrane, including the PERK-eIF2α-ATF4-CHOP, inositol-requiring enzyme 1 (IRE1)-X-box binding protein 1 (XBP1), and ATF6 pathways. PERK phosphorylates eIF2α and attenuates global mRNA translation, but it specifically induces ATF4 and CHOP. These factors regulate the expression of genes involved in restoring homeostasis and of GADD34 (together with PP1c, forms an eIF2α phosphatase complex), which feeds back to attenuate the response. IRE1, an endoribonuclease (RNase) with kinase activity, catalyzes the unconventional splicing of the transcription factor XBP1 mRNA, resulting in a potent transcription factor that upregulates genes for ER folding capacity expansion. ATF6 is translocated to the Golgi apparatus, where it is cleaved to release a cytosolic transcription factor that regulates chaperone expression and genes involved in ER-associated degradation [104] (Figure 4). These transmembrane sensors bind to the ER luminal chaperone GRP78/BiP. When a large portion of GRP78/BiP is sequestered by unfolded proteins, the sensors in the cytoplasm are activated via autophosphorylation, transducing signals to restore homeostasis.

The role of ATF4 in the PERK-eIF2α-ATF4-CHOP pathway has been extensively explored, and it plays a crucial role in cell death in vivo and ex vivo. In ER stress, ATF4 acts as a transcription factor. It binds to the promoters of C/EBP homologous protein (CHOP/DDIT3) and other target genes, inducing their expression to regulate protein folding, degradation, and stress adaptation. In mice with bleomycin-induced lung injury, high ATF4 expression induced in alveolar epithelial cells (AECs) exacerbated lung mitochondrial UPR (UPRmt), inflammation, body weight loss, and mortality, suggesting that ATF4 mediates UPRmt in response to mitochondrial stressors [105]. Glucose deprivation (GD) experiments revealed that PERK is the major kinase driving ATF4 activation, and activation of the ATF4 pathway represents a key downstream event of the UPR [106]. Vinigrol specifically activates the PERK arm of the UPR, leading to ATF4 and CHOP induction, which drives the UPR and results in non-apoptotic death of breast cancer cells [107]. The expression of ATF4 and FAM129A is clinically increased in prostate cancer patients, and therapeutic silencing of the ATF4-FAM129A axis inhibits tumor growth [108]. These data suggest that ATF4 modulates cancer development by regulating the UPR and has emerged as a potential therapeutic target.

## 7. ATF4 Regulation of Metabolic Reprogramming

### 7.1. Glucose Metabolism

**Figure 5:**
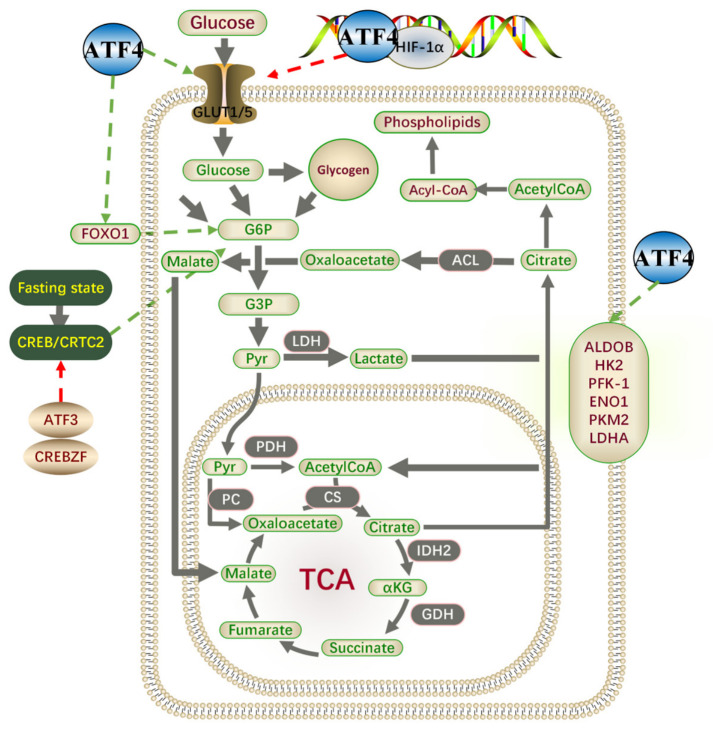
ATF4’s role in glucose metabolism and the TCA cycle. ATF4 transcriptionally activates GLUT1/5, enhancing glucose uptake into cells. Glucose is phosphorylated to G6P, which branches into glycogen storage or glycolysis. For glycolysis, ATF4 promotes expression of rate-limiting enzymes (such as HK2, PFK-1, PKM2) via HIF-1α, driving pyruvate production. Pyruvate is shunted to lactate by LDHA(anaerobic conditions) or channeled into the TCA cycle through PDH. Mitochondrial citrate (from TCA) is exported to the cytosol, where ACL converts it to acetyl-CoA (for lipid synthesis) and oxaloacetate. ATF4 also induces ALDOB, supporting TCA cycle flux and redox balance. In parallel, ATF4 represses gluconeogenic genes (PCK1) via ATF3/CREBZF. This network adaptively couples glucose utilization to energy production and biosynthesis during stress.

Cancer cell proliferation is usually driven by the inactivation of antioncogenes or activation of oncogenes, while post-tumorigenic alterations in cellular properties, such as aerobic glycolysis (later termed the “Warburg Effect”), have become a research focus [109,110]. ATF4 activation is detected in various cancers and contributes to the generation of anabolic precursors and energy via aerobic glycolysis [10,111,112] (Figure 5). Under normal physiological conditions, cellular metabolism is transcriptionally regulated by stress responses, including ISR [4]. ATF4 promotes the expression of glucose transporters glucose transporter type 1 (GLUT1)/GLUT5 and key metabolic enzymes, such as aldolase B and fructose-bisphosphate (ALDOB) (regulating glycogenolysis and glycolysis) [113,114]. In the glycolytic pathway, ATF4 knockdown downregulated the expression of enzymes, including phosphofructokinase-1 (PFK-1), enolase 1 (ENO1), pyruvate kinase M2 (PKM2), and lactate dehydrogenase A (LDHA) [115,116,117]. ATF4 directly binds to the CRE in the promoter of Hk2, the major HKs isoform that enhances the Warburg effect, thereby boosting glycolytic capacity [118,119]. The HIF-1α signaling pathway is a critical regulator of glycolysis, with ubiquitination-dependent stability control being essential for its activity. In hypoxic osteoblasts, ATF4 interacts with HIF-1α to induce its ubiquitination and degradation [118,120]. Additionally, ATF4 is pivotal in gluconeogenic homeostasis. Under fasting, the glucagon receptor-G protein-coupled receptor (GPCR) binds glucagon, stimulating cAMP production. PKA phosphorylates and activates CREB, which cooperates with CRTC2 to enter the nucleus. CREB/CRTC2 complex binds to CRE motifs in the promoters of gluconeogenic enzymes, such as Glucose-6-phosphatase (G6Pase) and phosphoenolpyruvate carboxykinase (PEPCK), while also inducing PGC-1α and PEPCK expression to enhance gluconeogenesis [121,122,123]. Other ATF family members (ATF3, CREBZF) inhibit glycolysis by disrupting CREB-CRTC2 interactions [124]. CREBH forms a complex with PPARα, facilitating gluconeogenesis (or glycolysis in specific tissues) via unique binding sequences distinct from CREB/CRTC2. Forkhead box protein O1 (FOXO1) is the master transcriptional activator of gluconeogenesis during prolonged fasting, regulating rate-limiting enzymes (G6Pase, PEPCK). ATF4 promotes FOXO1 expression, thereby regulating glucose homeostasis [125]. In HFD-fed middle-aged/elderly mice, ATF4 knockdown prevented pathological states like hyperglycemia, glucose intolerance, and obesity [126]. Furthermore, ATF4 mediates insulin resistance by inducing transcription of the Akt inhibitor tribbles homolog 3 (TRB3) [127].

### 7.2. Lipid Metabolism

ATF4-mediated lipid metabolism is related to cellular state and environment. ATF4-deficient mice showed a tendency to lower hepatic triglycerides (TG) and cholesterol content compared to WT mice. ATF4 deficiency increases expression of genes related to lipid catabolism, including hormone-sensitive lipase (*HSL*, mediating adipose tissue lipolysis), carnitine palmitoyltransferase 1 (*CPT1*), and medium-chain acyl-CoA dehydrogenase (*MCAD*, both involved in mitochondrial β-oxidation), thereby enhancing energy expenditure [128]. ATF4 stimulates the pro-adipogenic genes such as Sterol regulatory element-binding protein 1c (*SREBP-1c*) and *PPARγ*, along with their downstream lipogenic targets including acetyl-CoA carboxylase (*ACC*), fatty acid synthase (*FAS*), and stearoyl-CoA desaturase 1 (*SCD1*), to promote de novo lipogenesis and induce hepatic steatosis [129,130,131]. Notably, ATF4 also binds to the AARE motif (assumed to be a specific response element in *FGF21* regulation) in the promoter of the hepatokine *FGF21*, increasing its expression in the livers of high-fat diet (HFD)-induced ATF4-deficient mice. This upregulates *FGF21*, which in turn reduces hepatic TG and ameliorates steatosis [132,133,134,135]. In addition, using promoter assays, immunoblotting, and siRNA analyses demonstrate that ATF4 activation mediates the transcriptional activity of sphingosine kinase 2 (*SphK2*) [136].

### 7.3. Amino Acid Metabolism

Glutamine is the most abundant amino acid in human plasma and a critical nitrogen donor for nucleotide/NEAA biosynthesis. Along with glucose, it is a major contributor to gap-filling fluxes [137]. ATF4 facilitates catabolism such as glycolysis and glutaminolysis, whose products provide precursors for amino acid synthesis. First, ATF4 upregulates glycolytic genes to increase pyruvate production (via glycolysis), which enters the TCA cycle to supply carbon/energy for amino acid synthesis and T cell growth. Second, ATF4 upregulates *GPT2* (alanine aminotransferase, ALT), which catalyzes the reversible transamination of L-glutamate and pyruvate to α-ketoglutarate and L-alanine, indirectly supporting TCA cycle anaplerosis. Additionally, ATF4 transcriptionally activates the glutamine transporter solute carrier family 1 member 5 (*SLC1A5*) to enhance glutamine uptake [138]. Thus, ATF4 promotes anaerobic flux by enhancing glutamine catabolism. Since TCA cycle metabolites, such as citrate, OAA, and α-ketoglutarate, are amino acid precursors, ATF4 increases amino acid anabolism by promoting anaplerotic responses [139]. In addition, ATF4 upregulates key amino acid metabolic enzymes, such as *ASNS*, argininosuccinate synthase 1 (*ASS1*), *SLC1A5*, phosphoglycerate dehydrogenase (*PHGDH*), elevating intracellular amino acid levels [140,141,142].

## 8. ATF4 Regulation of Autophagy

Autophagy is a major cellular self-protection mechanism that allows stressed cells to survive by recycling macromolecules (including protein aggregates and old or damaged organelles) and restoring metabolic homeostasis. In addition to the ubiquitin–proteasome system, oxidative stress, proteotoxicity, and metabolic stress increase autophagy and help restore homeostasis. Autophagy requires the concerted involvement of a set of proteins involved in the formation of autophagosomes and autolysosomes, as well as cargo-selective proteins that direct the degradation [143,144]. ATF4 binds to the AARE or related CRE-like sequences in the promoters of its target genes, thereby orchestrating diverse transcriptional programs. Among these targets are genes involved in autophagy, such as *Map1lc3b (lc3)* and autophagy related 5 (*Atg5*), leading to autophagosome formation and activation [145,146]. ATF4 binds to the promoter AARE sequence of autophagosomes and triggers various types of transcriptional responses in different stress contexts, inducing autophagosome formation and activation [147]. Autophagy genes activated by ATF4 can be broadly classified into three groups: (I) proteins encoding the ubiquitin-like protein (Ubl) system, including four Ubl proteins (*Map1lc3b*, *Gabarap*, *Gabarapl2* and *Atg12*), an activating enzyme (*Atg7*), the target of Atg12 attachment (*Atg5*) and *Atg16l1*; (II) proteins encoding autophagosome formation and maturation such as *Becn1*; (III) cargo receptors encoding specific degradation of ubiquitinated substrates such as *p62* and *Nbr1* [147]. In addition, *Map1lc3b* and *Atg5* were also identified as ATF4 targets through the PERK/eIF4α pathway [145]. Interestingly, during autophagy activation, the relevant autophagy proteins are continuously degraded upon fusion with lysosomes. ATF4 may have a key role in complementing degraded proteins to enable the cell to maintain the autophagic state.

## 9. ATF4 Regulation of Inflammation and Immunity

ATF4 is widely expressed across diverse immune cell types, such as monocytes, T cells, B cells, natural killer cells (NK cells), macrophages, dendritic cells, and myeloid-derived suppressor cells (MDSCs), suggesting its immunomodulatory role in systemic immune regulation [148]. Infection or depletion of innate immune cells triggers inflammation, a dynamic process encompassing injury, anti-injury, and repair that modulates the balance of physiological and pathological signaling networks and exhibits a “high degree of duplicity” [149,150,151,152]. Under conditions of persistent/low-intensity stimuli or prolonged/target tissue overreaction, inflammation fails to transition from an anti-infective/tissue-damaging mode to a balanced steady state, resulting in a persistent inflammatory response that generates excess ROS and progresses to chronic inflammation. Chronic inflammation is a root cause of numerous age-related diseases, including cardiovascular disease, diabetes, cancer, and Alzheimer’s disease [153,154,155].

### 9.1. ATF4 Exerts Bidirectional Regulation of Inflammation

**Figure 6:**
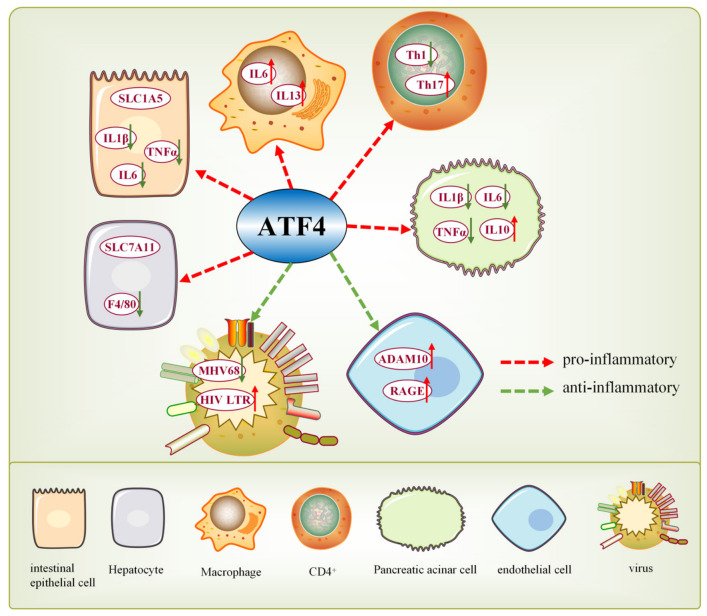
The dual regulatory roles of ATF4. It promotes pro-inflammatory (red dashed arrows) and anti-inflammatory (green dashed arrows) responses, across diverse cell types and viral infection contexts.

Multiple studies confirm ATF4’s dual role in inflammation: it can suppress or promote immune responses in a cell type- and disease-dependent manner (Figure 6). Patients/mice with IBD show reduced ATF4 expression. ATF4 knockout induces spontaneous small intestinal colitis in IEC mice and enhances susceptibility to dextran sodium sulfate (DSS)-induced colitis [156]. ATF4 knockout increases the susceptibility of normal hepatocytes to ferroptosis in mice and enhances the hepatic inflammatory response in mice, thus promoting compensatory liver proliferation and hepatocellular carcinoma (HCC) [6]. In addition, ATF4 blocks inflammation by directly inhibiting the transcription of pro-inflammatory cytokine genes or suppressing the activity of NF-κB signaling and the expression of its upstream factors, including Toll-like receptor 4 (TLR4). In GRP78-deficient mice, high levels of *ATF4* mRNA increases IL-6 expression, polarizing them to the M2 state and stimulating IL-13 signaling/skeletal muscle glucose metabolism [157]. Silencing of ATF4 in pancreatic follicular cells reduces pro-inflammatory proteins (IL-1β/IL-6/TNF-α) and increases the anti-inflammatory protein IL-10 [158]. ATF4 deficiency reduces Th1 responses and enhances Th17 responses in vivo (via reduced Th1 differentiation driving Th17 phenotype) [139]. In LPS-induced inflammation, ATF4 silencing inhibits IL-6/IL-8 transcription/secretion and reduces THP-1 cell adhesion to HUVECs [159].

ATF4 also promotes inflammation or fine-tunes immunity through post-translational regulation and viral immune evasion. ATF4 modulates ADAM10 to induce shedding of advanced glycation end product (AGE) receptors [160]. B cell receptor (BCR) signaling inhibits ATF4 to trigger C/EBP homologous protein (CHOP), promoting lysogenic replication of murine gammaherpesvirus 68 (MHV68). Simultaneously, ATF4 inhibits MHV68 replication and promoter activity of its replication and transcription activator (RTA, a cleavage-switch transactivator), regulating the infection cycle [161]. Acute simian immunodeficiency virus (SIV) infection enhances protein synthesis inhibition and GCN2-ATF4 signaling, whereas this inhibition and activation were attenuated during chronic viral infection. In CD4^+^ T cells, ATF4 binds to the HIV long terminal repeat (LTR) and promotes viral transcription. Enhanced and inhibited ATF4 expression was found to reactivate HIV and inhibit HIV expression [162]. ATF4-deficient CD4^+^ T cells exhibit defects in redox homeostasis, proliferation, differentiation, and cytokine production [139]. Thus, the complex process of viral evasion of immune detection may involve the regulation of T cells by ATF4.

### 9.2. ATF4 in Cancer Immunity: Balancing Immune Surveillance and Evasion

During cancer progression, ATF4 modulates immune cell function to either suppress tumors or enable immune escape. ATF4 suppresses the effector function of CD8^+^ T cells. T cell-specific CHOP deletion enhances anti-tumor CD8^+^ T cell immunity and improves T cell-based immunotherapy efficacy. Sustained ER stress upregulates CHOP via ATF4, which directly inhibits T-box transcription factor *Tbet* expression—forming a ATF4-CHOP negative regulatory pathway for CD8^+^ T cell effector function [163]. Meanwhile, MDSC suppresses immune activity, mainly by targeting T cells. MDSC can be classified into two types, M-MDSC (antigen-specific and non-specific modality) and PMN-MDSC (antigen-specific modality), both exerting inhibitory effects on T cell responses [164]. ATF4 interacts with MDSCs to modulate their activity. ROS/nitrogen species drive ATF4-mediated CHOP expression in MDSCs, promoting CHOP accumulation and immunosuppressive activity [165,166]. CHOP-deficient MDSCs reduce IL-6 production and phosphorylated STAT3 expression [167]. Myeloid GCN2 deletion (which limits ATF4 translation) drives MDSC transformation to promote anti-tumor immunity [167], but GCN2-ATF4 signaling also enhances MDSC activation and CD8^+^ T cell *IFN-γ* expression, worsening the tumor microenvironment [168]. Thus, ATF4 exerts its immunomodulatory role by regulating the overall activity of MDSCs. In addition, NK cells are part of the natural immune response and function as a defense against viral infections and tumors in the absence of any initiation. Forward genetic screening identifies ATF4 as a critical regulator of *ULBP1* transcription—ULBP1 is a ligand for the natural killer group 2 member D (NKG2D) receptor. ATF4 knockdown significantly reduces ULBP1 transcription, suggesting its role in driving NK cell-mediated immune responses [169].

ATF4’s immunomodulatory role is defined by context-dependent bidirectional regulation: it integrates stress signals to modulate inflammation, viral immunity, and cancer surveillance via direct/indirect control of immune cell maturation, polarization, and function. Its broad impact on effector immune cell dysfunction highlights its potential as a therapeutic target for immunotherapy. However, deeper analysis of ATF4’s role in microenvironmental tissue-specific regulation is needed to fully exploit its clinical value.

## 10. ATF4’s Roles in Diseases

### 10.1. Cancer

ATF4 usually acts as an oncogene, mainly functions through metabolic reprogramming, resistance to oxidative stress and ferroptosis, and synergises with oncogenic signaling pathways. N-acetyltransferase 10 (NAT10) enhances *ATF4* mRNA stability through N4-acetylcytidine modification. ATF4 then transcriptionally activates *ASNS*, promoting asparagine synthesis, ultimately driving the progression of osteosarcoma [170]. In human T cell acute lymphoblastic leukemia (T-ALL), ATF4 drives the expression of *ASNS*, *ASS1*, and *PHGDH*, thereby maintaining mTORC1 activation [171]. ATF4 binds to the CARE motif located in the promoters of fructolysis genes (*SLC2A5*/*ALDOB*), shifting energy metabolism from glycolysis to fructolysis to adapt to glucose deprivation, ultimately promoting the growth of glioblastoma multiforme (GBM) [114]. Additionally, in cancers such as Pancreatic ductal adenocarcinoma (PDAC), esophageal squamous cell carcinoma (ESCC), colorectal cancer (CRC), liver cancer, and renal cell carcinoma (RCC), the ATF4-SLC7A11-GSH axis is a key pathway for tumor cells to resist oxidative stress and maintain proliferation [172,173,174,175,176]. ATF4 also cooperates with JMJD6 to activate *SLC7A11*, *GCLM*, and *ME1* in prostate cancer [177]. Oncogene MYC activates the GCN2/ATF4 pathway. ATF4 collaborates with MYC to upregulate specific target genes (particularly *4E-BP1*), thereby balancing MYC’s synthetic drive, alleviating proteotoxic stress, ultimately ensuring cell survival under high MYC activity and promoting tumor progression [22].

ATF4 also has tumor-suppressive functions. Our previous study demonstrated that, although ATF4 expression is upregulated in established HCC, in normal hepatocytes ATF4 exerts a tumor-suppressive role by activating its target gene *SLC7A11* (encoding the xCT transporter subunit) to sustain glutathione synthesis, thereby inhibiting ferroptosis, alleviating inflammation and liver injury, and suppressing compensatory proliferation [6]. The natural compound Halofuginone suppresses *COL1A1* expression by antagonizing the mTOR-eIF2α-ATF axis, thereby enhancing chemosensitivity, indicating that reduction in ATF4 plays a beneficial role in ovarian cancer [178]. Upon s-phase kinase associated protein 2 (SKP2) and phosphatase and tensin homolog (PTEN) inactivation, ATF4 induces cellular senescence via the upregulation of cyclin-dependent kinase inhibitors *p21* and *p27*, thereby restricting tumorigenesis [179]. In conclusion, ATF4 plays a highly context-dependent dual regulatory role in cancer, functioning like a “double-edged sword”—it can act as a key oncogenic factor driving tumor progression, while also inducing protective responses under specific genetic contexts to restrict tumorigenesis.

### 10.2. Cardiovascular Diseases

Taurochenodeoxycholic acid (TCDCA) activates the farnesoid X receptor (FXR) on vascular endothelial cells, then inhibits PHB1 protein and thereby releases ATF4. ATF4 then directly binds to and promotes the transcription of *PHGDH*, *PSAT1*, *PSPH*, *SHMT2*, and *MTHFD2*, restoring vascular NO bioavailability and reducing oxidative stress, ultimately improving vascular function [180]. ATF4 also promotes the development of hypertension. ATF4 deficiency prevents high-salt diet-induced hypertension in mice and protects endothelial function [42]. The high-salt diet directly increases ATF4 expression levels in vascular endothelial cells. Elevated ATF4 causes hyperactivity of calcium-related proteins (such as CaMK4, CACNA1C, IP3R, and RyR2), reduces GPX4 level, and upregulates key enzymes that promote lipid peroxidation (such as ACSL4 and LPCAT3) [181]. In atherosclerosis, ISRIB-mediated inhibition of ATF4 promotes plaque stabilization by improved macrophage DNase release and neutrophil extracellular traps (NETs) degradation [182]. ATF4 directly binds to the promoter region of *AIM2* inflammasome and promotes its transcription. Activated AIM2 inhibits ABCA1 expression (a key transporter mediating cholesterol efflux to apolipoprotein A-I), leading to reduced cholesterol efflux and foam cell formation [183]. Given the complex dual role of ATF4 in cardiovascular diseases, future therapies must tailor its activation or inhibition based on disease stage and cellular state for optimal outcomes.

### 10.3. Fibrosis

HSC-specific ATF4 knockout inhibits liver fibrosis in mice, and human HSC ATF4 levels correlate positively with fibrosis progression. ATF4 serves as an epigenetic hub that activates enhancer programs to drive EMT-related genes [184]. ATF4 is overexpressed in idiopathic pulmonary fibrosis (IPF) patients and animal models, and its blockade attenuates bleomycin-induced pulmonary fibrosis. ATF4 directly binds to the promoter of *ACTA2* (encoding α-SMA), promoting lung fibroblast activation and myofibroblast accumulation. Moreover, ATF4 regulates the macrophage M2 program and enhances TGFβ1 secretion [185]. ATF4 binds the *PPARGC1A* promoter to drive FAO metabolic reprogramming and reverse progressive lung fibrosis [186]. ATF4 upregulates hexokinase II (HK-II) to drive glycolysis in renal tubular epithelial cells, thereby promoting tubulointerstitial fibrosis [187]. In diabetic nephropathy, high ATF4 expression inhibits protective autophagy [188]. In diabetic cardiac fibrosis, ATF4 drives Smurf2-mediated HO-1 degradation, exacerbating fibrosis and oxidative stress [189]. Overall, ATF4 is a key pro-fibrotic hub. Its pro-fibrotic functions are primarily achieved through transcriptional regulation and metabolic reprogramming independent of ER stress, notably in the liver, lung, and kidney.

### 10.4. Other Diseases

In Parkinson’s disease, ATF4 knockdown is neuroprotective by directly inducing pro-apoptotic *CHOP*/*TRIB3*/*PUMA* to drive dopaminergic neuron loss [190]. Downregulation of ATF4 alleviates ER stress (CHOP/GADD-34), neuroinflammation, and cognitive impairment, positioning ATF4 as a potential therapeutic target for Alzheimer’s disease [191]. ATF4 promotes the expression of key lipogenic genes (such as *PPARγ* and *SREBP-1c*), and ATF4-deficient mice are resistant to high-fructose-induced hypertriglyceridemia and hepatic steatosis [131]. Furthermore, upon the Piezo1/β-catenin/ATF4 axis being impaired, ATF4 function is suppressed, impairing Gli1 BMSC proliferation and osteogenic differentiation, thereby leading to bone loss and osteoporosis [192].

## 11. Discussion

ATF4 is an important downstream factor of ISR/ER, which further regulates the expression of its target genes and exerts a wide range of orchestrated functions in a variety of diseases, especially metabolism or inflammation-related diseases. So, understanding ATF4-mediated metabolism and immunity in different cell types in the context of disease will help to discover new therapeutic strategies in molecular pathology.

The cell type- and context-dependent functions of ATF4 are a hallmark feature, yet the underlying molecular switch mechanisms remain unclear. For example, ATF4 can exhibit both pro- and anti-tumorigenic roles in the same cancer type (liver cancer), but the precise nodes governing this duality—such as specific post-translational modification (PTM) combinations, dynamic choice of dimerization partners, or altered subcellular localization—remain undefined. Moreover, the complexity of the ATF4 dimerization network exceeds current knowledge. ATF4 forms homo- or heterodimers with multiple bZIP members; however, how distinct dimers selectively recognize target gene promoter elements (CARE, ARE, AARE) and how this selectivity is remodeled by stress intensity, duration, or metabolic state is unknown. Furthermore, the spatiotemporal interplay of ATF4 PTMs (phosphorylation, acetylation, hydroxylation, ubiquitination)—and their fine regulation of protein stability, transcriptional activity, and protein–protein interactions—lacks systematic dynamic analysis. No direct and specific ATF4-targeting drugs are currently approved. Given its pro-tumorigenic role in most cancers, small-molecule inhibitors that block ATF4 transcriptional activity or protein stability are worth developing. The reported compound ISRIB (which indirectly downregulates ATF4 via inhibition of the eIF2α phosphatase complex) has shown promise in liver cancer, neurodegenerative diseases, and pulmonary fibrosis [193,194,195]. In addition, inhibitors targeting the upstream kinase GCN2 (APL-4098) [196] or the downstream effector *ASNS* (Bisabosqual A) [197] have demonstrated anti-cancer effects in preclinical studies. However, more selective direct inhibitors or activators of ATF4 are urgently needed.

In conclusion, ATF4 is a key molecule at the intersection of stress adaptation, metabolic regulation, and immune surveillance. Future research should integrate multidisciplinary approaches—ranging from molecular mechanisms to in vivo models—to systematically decipher the fine-tuned regulatory logic of ATF4. On this basis, spatiotemporally controllable and tissue-specific interventions should be developed to translate fundamental discoveries on ATF4 into effective therapeutic targets in precision medicine.

## Figures and Tables

**Table 1:** miRNAs that regulate ATF4.

miRNA	Regulation Direction	Cell/Disease Models	Reference
miR-214	Inhibits ATF4	Osteoporosis, thalassemia, hepatocellular carcinoma	[39,40,41]
miR-1283	Inhibits ATF4	Hypertension	[42]
miR-383-3p	Inhibits ATF4	Myocardial ferroptosis	[43]
miR-193a-3p	Indirectly inhibits ATF4	Bone remodeling	[44]
miR-93-3p	Indirectly inhibits ATF4	Stress urinary incontinence	[45]
miR-129/342	Indirectly inhibits ATF4	Calcified aortic valvular	[46]
miR-15b-5p	Indirectly promotes ATF4	Cell ferroptosis	[47]
miR-3200-5p	Inhibits ATF4	Hepatocellular carcinoma	[48]
miR-663	Promotes ATF4	Atherosclerotic	[49]
miR-320a	Indirectly promotes ATF4	Human colon cancer cells, mouse embryonic fibroblasts	[50]
miR-4734	Promotes ATF4	Human foreskin fibroblasts	[51]

## Data Availability

No new data were created or analyzed in this study. Data sharing is not applicable to this article.

## Abbreviations

The following abbreviations are used in this manuscript:

ATF4	Activating transcription factor 4
ER	Endoplasmic reticulum
ISR	Integrative stress response
eIF2α	Eukaryotic initiation factor 2 subunit alpha
CREB	cAMP-response element binding
CREM	cAMP-responsive element modulator
BD	Basic domain
BTZ	Bortezomib
PHD3	Prolyl-4-hydroxylase domain 3
ODD	Oxygen-dependent degradation
βTrCP	β-transducin repeat-containing protein
PCAF	p300/CBP-associated factor
UPR	Unfolded protein response
CK1δ	Casein kinase 1δ
PKA	Protein kinase A
CLK2	Cdc-like kinase 2
PRMT5	Protein arginine methyltransferase 5
PDX1	Pancreas-specific transcription factor
NUPR1	Nuclear protein 1
uORF	Upstream open reading frame
BCL-2	B-cell lymphoma-2
MCL-1	Myeloid cell leukemia-1
C/EBP	CCAAT/enhancer binding protein
HSPA5	Heat shock protein family A member 5
GLUT1	Glucose transporter type 1
GPCR	G protein-coupled receptor
HSL	Hormone-sensitive lipase
CPT1	Carnitine palmitoyltransferase 1
MCAD	Medium-chain acyl-coenzyme A dehydrogenase
ACC	Acetyl coenzyme A carboxylase
NEAA	Non-essential amino acid
SLC1A5	Solute carrier family 1 member 5
GD	Glucose deprivation
MDSCs	Myeloid-derived suppressor cells
IBD	Inflammatory bowel disease
DSS	Dextran sodium sulfate
LTR	Long terminal repeat
